# Genital warts and women’s sexual challenges: is sexual compatibility possible? A qualitative study

**DOI:** 10.1186/s12905-023-02771-9

**Published:** 2023-11-16

**Authors:** Mohadese Adeli, Lida Moghaddam-Banaem, Shadab Shahali, Tahereh Soori

**Affiliations:** 1 Department of Reproductive Health and Midwifery, Faculty of Medical Sciences, Tarbiat Modares University, Tehran, Iran; 2 Department of Midwifery, School of Nursing and Midwifery, Torbat Heydariyeh University of Medical Sciences, Torbat Heydariyeh, Iran; 3 Department of Infectious Disease, Arash Women’s Hospital, Tehran University of Medical Sciences, Tehran, Iran

**Keywords:** Genital warts, Human papillomavirus, Sexual dysfunction

## Abstract

**Background:**

Genital warts, besides their importance as symptoms of sexually transmitted infections, can also threaten the sexual health of couples. The purpose of this study was to explore the sexual compatibility in women with active genital warts.

**Methods:**

A qualitative study with a conventional content analysis approach in Tehran, Iran, from January 2019 to February 2020 was conducted on a purposeful sample of data saturation achieved after interviewing 14 women with genital warts, 2 couples and 3 dermatologists, 1 sexologist and 1 infectious disease specialist. Data were collected through unstructured interviews and analyzed using conventional content analysis approach.

**Results:**

After data analysis 224 initial codes, 5 main categories “change in the frequency of sexual activity”, “changing types of sexual intercourse”, “protected sexual intercourse”, “concealment of lesions”, “focusing on personal hygiene by couples after sexual activity” and finally a central theme of “adaptation to challenges of sexual intercourse” were extracted.

**Conclusions:**

This study revealed the perceptions and experiences of women with active genital warts about the process of their sexual adaptation. The main concepts found in this study focus on challenges related to sexual intercourse. It seems that recognizing women’s sexual adaptation challenges after getting genital warts may help them take effective and practical measures to improve their sexual compatibility and health.

## Background

Genital warts (condyloma acuminate) are the clinical manifestations of a sexually transmitted infection caused by some types of human papillomavirus (HPV) [1].

The age of onset of genital warts in men is 25–29 years old and in women 20–24 years old [2–4]. In Iran, accurate information on the prevalence of genital warts is not available, and most studies have reported only the prevalence of HPV; the prevalence of HPV in Tabriz was 20.8% [5] and in Tehran 5.7% (2008) and 7.8% (2012) [6, 7].

The physical symptoms of active genital warts can include itching, burning, and pain around the affected area. They may also cause bleeding and discomfort during sex. In some cases, genital warts can lead to more serious health issues, such as cervical cancer [8].

In addition to the physical symptoms, active genital warts can also have a significant emotional and psychological impact on women. They can cause feelings of shame, embarrassment, and anxiety, as well as social isolation and relationship problems [9, 10]. Also, Genital warts reduce the quality of life by changing daily activities, it also causes pain and discomfort, anxiety, and depression [8]. Women with genital warts experience more severe psychosexual side effects, including emotional, sexual life, negative self-image, self-esteem, interaction with a doctor and fear of transmitting the disease to a sexual partner [9, 11].

Sexual compatibility has been described as mutual understanding and adjustment in emotional, cognitive, and behavioral factors during sexual encounters. In other words, the maturity of couples in psychological and interpersonal relationships increases satisfaction with sexual intercourse [12]. In terms of importance, sexual compatibility is in the first line of a healthy married life [14]. According to the results of Nekulal et al. (2017) qualitative study, sexual compatibility in Iranian couples means the couple’s participation in meeting each other’s sexual needs and solving problems caused by sexual incompatibilities. Sexual incompatibility is inevitable in sexual relationships, but compatible couples face it based on understanding, agreement, and love [15]. Sexual compatibility in couples increases the satisfaction of the sexual relationship [15–17]. On the other hand, sexual incompatibility can lead couples to divorce [18, 19]. Evidence shows that 50–60% of divorces are because of the sexual relations of couples [20–22].

The passage of time in married life may decrease sexual compatibility. However, sexual compatibility has been reported to be related to couples’ emotional, psychological, and interpersonal maturity [12].

Sexual compatibility has been associated with depression and anxiety. Women with higher sexual adjustment scores have less depression and report higher levels of motivation and sexual desire [23]. The relationship between sexual adjustment and depression and anxiety has been reported to be stronger in women than in men [24].

Sexually transmitted diseases jeopardize couples’ sexual compatibility. Studies have confirmed that in people with HPV, one of the most common sexually transmitted diseases, genital warts have the most negative impacts on couples’ sexual life [25, 26]. There are a few studies about sexual dysfunction in women with genital warts, but no study has been found to assess how these patients deal with sexual incompatibility caused by active genital warts, and unless it is clear to patients how to deal with these problems, it is impossible to provide an appropriate treatment strategy. Therefore, this study was conducted to explore the compatibility and adaptation of women with active genital warts to sexual activity.

## Materials and methods

This qualitative study was conducted in the Dermatology Clinic of Razi Hospital in Tehran, Iran, from January 2019 to February 2020. In order to select a sample with rich experience, a purposeful sampling method was used to select women or couples who had experience of living with genital warts and specialists with experience of treating the sexual dysfunction of these patients.

Inclusion criteria were: Aged 18 or over, having sexual activity, having been diagnosed with genital warts by a gynecologist, dermatologist, or infectious disease specialist, with no history of genital malignancy. Inclusion criteria for the specialists were: the history of providing services to women with genital warts, and willingness to participate in the study.

The exclusion criteria included women who had a history of HPV vaccination, were suffering from other sexually transmitted diseases, physical, mental and sexual diseases (affecting psycho-social and sexual health) and diseases of the immune system, or were consuming alcohol, drugs and tobacco.

Prior to data collection, permission to enter the clinical area was obtained from the research Ethics Committee of Tarbiat Modares University (supplementary file 1) and the relevant hospital officials after the Research Council of the Faculty of Medical Sciences approved the proposal. This study has been performed in accordance with the Declaration of Helsinki, all ethical principles in research such as obtaining informed consent, anonymity, confidentiality, and participants’ right to withdraw from the study were followed. Unstructured in-depth individual interviews were used for data collection. All interviews were conducted at the Dermatology Clinic of Razi Hospital in Tehran and in a private room according to the participants’ preference. The questions as open questions, general questions and more specific questions such as “How has the diagnosis and treatment of genital warts affected your sexual life? In-depth questions based on the information provided by the participant were asked to obtain more detailed information and to clarify the concept under study, such as “What does your statement mean? Can you explain what you mean with an objective example? The average duration of each interview was 60 minutes. It should be noted that all interviews were conducted before the Covid-19 pandemic, and only one interview with a sexual health specialist was conducted online after the Covid-19 pandemic. Data collection continued until data saturation was reached. With the consent of the participants, all interviews were audio-recorded. The interviews were then transcribed verbatim. Nonverbal gestures and postures were also noted and transcribed into Word. In addition to the interviews, field notes were used in the data collection process, while a conventional qualitative content analysis was conducted following Graneheim & Lundman’s (2004) steps [21]. First, after immersion in the data, the researcher read the typed texts several times to become familiar with the text of the data. After determining the semantic units of phrases, sentences, and paragraphs, the primary codes were extracted. Similar primary codes were grouped into subcategories, and similar subcategories were grouped into main categories. In addition, some subcategories were recoded and renamed. Finally, the theme was extracted from the main categories. To ensure the trustworthiness of the results, four Lincoln & Guba criteria were used [22, 23].

## Results

To increase the accuracy of the data, we tried to interview women with genital warts who had the greatest variation in age, education, occupation, duration of marriage, and duration of disease. Their demographic characteristics varied in terms of education from middle school to bachelor’s degree, in terms of duration of marriage from 1 to 28 years, and in terms of duration of illness from 1 month to 3 years based on age from 18 to 55 years.

The demographic characteristics of the spouses of these patients also include the average age of 42 years, the average duration of marriage of 13 years, and the level of high school education. An attempt was made to interview the specialists who had the most contact with these patients. In this regard, we spoke with gynecologists and reproductive health specialists who said that these patients would be referred to a dermatologist or infectious disease specialist if they were referred to them. Therefore, only dermatologists, sexologists, and infectious disease specialists were interviewed. The average age of the group of specialists was also 43 years, and their average work experience was 12.25 years.

After conducting 23 interviews with 23 participants about understanding and experiencing sexual compatibility after genital warts (16 women, 2 spouses, and 5 specialists), 224 primary codes were extracted from the quotes. For example, a participant’s words such as “We consulted the doctor and she advised us not to have sexual intercourse until the treatment is completed (MS P3, 24 years old, housewife, duration of marriage: 7 years)” led to the creation of the primary code Avoiding sexual intercourse because of the doctor’s advice.

The primary classification of codes began with the first interviews to form classes and subclasses. In subsequent interviews, the codes from each interview were compared to each other and to other codes from previous interviews to determine their similarities and differences. The codes were then placed into a specific subcategory based on the similarities they had with each other.

For example, the codes of avoiding sexual intercourse because of the doctor’s advice, avoiding sexual intercourse because of the fear of transmitting the disease to the spouse were placed in a subcategory because of their similarity to each other, and the name of avoiding sexual intercourse was chosen for it.

Similarly, the codes for reducing the number of sexual intercourse due to doctor’s advice, reducing the number of sexual intercourse due to fear of transmitting the disease to spouse, and reducing the number of sexual intercourse due to decrease in patient’s libido were placed in a subcategory because of their similarity to each other, and the name reducing the number of sexual intercourse was chosen for it.

Coding continued in the above manner for all interviews, and finally, based on the participants’ experiences, overt and hidden concepts were identified, and then these concepts were classified into 224 primary codes and 13 subcategories. The categories were reviewed and compared several times. This had a significant impact on the development of the categories and placing the codes in the appropriate categories, and resulted in some categories being merged or newer categories being created through further data collection and analysis. At the end of this phase, the primary codes were grouped into 13 subcategories, 5 major categories, and one theme. The abstraction process of this theme is presented in Table 1.

**Table 1:** Theme abstraction process

Theme	Category	Subcategory
Adaptation to challenges of sexual intercourse	change in the frequency of sexual activity	avoiding sexual intercourse
		Reducing the number of sexual intercourse
	change in the type of sexual intercourse	Sex without penetration
		vaginal sex
		avoiding oral sex
		avoiding anal sex
		Reducing anal sex
		Masturbation
		extramarital relationship
	Protected sexual intercourse	Use a condom
		Wearing underwear during sexual intercourse To reduce skin contact
	concealment of lesions	Covering lesions with Band-Aids during sexual intercourse
		Rapid treatment (cryo) to remove the lesions before having sexual intercourse with the husband
	focusing on personal hygiene by couples after sexual activity	Observance of personal hygiene immediately after sexual activity due to the belief that the source of infection is the lack of hygiene of the couple.
		Observing personal hygiene immediately after sexual activity to prevent the transmission, spread and recurrence of lesions

**Adaptation to challenges of sexual intercourse** This theme suggests women experience changes in sexual behavior after realizing genital warts. Some of these changes occur at the request of the patient or spouse, or after obtaining information from the Internet and other sources. Some of these behavioral changes are advised by doctors, that despite the restrictions on sex, couples are required to comply with it.

### Change in the frequency of sexual activity

change in the frequency of sexual activity is one of the dimension of **adaptation to challenges of sexual intercourse** so that, most patients spoke about a decrease in the number of sexual intercourses following knowing about genital warts:

“Before the disease, we had sex once or twice a week, but now it occurs once a month.” (P3).

Sometimes this decrease in the number of sexual intercourses occurs early in course of the disease, which improves over time:

“Yes, in the beginning, the number of our sexual intercourses was decreased. But now, it is not like that. Our sexual intercourse is the same as before.” (P11).

Sometimes, reducing the number of sexual intercourses was requested by the patient and the husband accompanied the patient in order to obtain the patient’s consent:

“My husband understands me. I think that despite his wishes, he has reduced the number of our sexual intercourses because of me.” (P8).

Sometimes the decrease in the number of sexual intercourse after the infection was the decision and desire of both couples, and the most important reason for them was preventing re-transmission of lesions to each other:

“We both agreed on it. My husband says it is better to wait until we get better and all the wart lesions should be removed so that, we do not transfer them again.” (P13).

Of course, fear of transmitting the disease to the spouse during sexual intercourse was one of the most important concerns of patients, which led to a decrease in the number of sexual intercourse after infection:

“The number of our sexual intercourse has decreased. It occurs rarely. Because, my skin has been involved and certainly, if we have sex, it will be transmitted to my husband.” (P3).

Fear of transmitting the disease in some patients was so great that they believed that having sexual intercourse with a condom is also likely to transmit the disease and thus having sex should be avoided until the lesions are removed. A participant stated:

“Others have told us it is better not to have sexual intercourse with a condom because; there is still the possibility of transmission.” (P7).

Doctors also advised patients to reduce or avoid having sexual intercourse until the end of treatment period, which was accepted by the couple most times:

“Because, we consulted the doctor. He told us not to have sexual intercourse at the moment because; there is a possibility of transferring from me to my husband. That is why we do not have sexual intercourse at the moment.” (P3).

Specialists also confirmed this claim, as a participant who was a dermatologist stated that:

“We usually recommend the patients not to have sexual intercourse until complete healing of lesions and at least by 6 months later ……. Frequent and excessive sexual contact should be reduced.” (P20).

### Change in type of sexual intercourse

Another dimension of Adaptation to challenges of sexual intercourse found in the present study was the type of sexual intercourse that can be influenced by genital warts. Most of the patients stated that after becoming aware about the disease, they tried to change some types of sexual intercourse. One of the most important changes mentioned was avoiding having oral sex, which almost all participants talked about it, and their most important reason was fear of transmitting the virus to the throat and larynx and developing laryngeal cancer.

“We have avoided having oral sex. I am scared. I also saw a movie about wart lesions in the throat. “I am afraid of developing warts in my throat.” (P11).

Some spouses, despite dissatisfaction with elimination of oral sex after the infection, responded to the patient’s wishes and accepted this change in sexual behavior.

“My husband used to insist on having oral sex. But because of me, he agreed to eliminate this type of sex. Now, he says nothing and does not complain, but I know he likes it to be so.” (P16).

Anal sex was another kind of sexual relationship that many patients reported that they have eliminated or reduced this type of sexual relationship after the infection. For example, one participant strongly criticized anal intercourse, and introduced it as the main cause of developing genital warts.

“Actually, I think these warts come from anal intercourse because, in the recent months, my husband wanted anal intercourse, after which I got infected.” (P6).

Fear about increasing and worsening of lesions was another reason cited by a participant who has avoided having anal sex after the infection:

“In the past, I only had anal sex occasionally, but I am no longer willing to have such intercourse ……. I am afraid of increasing and worsening of lesions.” (P12).

Some other participants had sex with no penetration after realizing the infection:

“After getting infected, we have not had vaginal penetration and tried to satisfy each other without penetration and by making love and touching each other.” (P6).

Participants cited different reasons for choosing this type of sexual relationship, which was one of the most important reasons for the doctor’s recommendation:

“Because, the doctor told us it is better not to have sex until the lesions are healed, thus, we did not have sex but, we have superficial sex without vaginal penetration.” (P7).

Elimination of sexual intercourse with penetration for any reason, in some cases caused dissatisfaction of the patient or spouse and reduced sexual satisfaction. A participant said that:

“It’s been an entire month now and we’ve been through some tough times, but we’re still stuck dealing with this restriction. I’m really hoping it’ll be over soon because we’re all pretty unhappy with how things are going.” (P6).

Masturbation was another dimension of changing sexual behaviors. Some patients commented on this. Some participants who had stopped having sex and were content with just flirting stated that they had masturbated during their disease.

“I am not sure about masturbation by my husband. I do not know that if he has masturbated, but I did it myself.” (P7).

Extramarital relationship is another subcategory of Adaptation to challenges of sexual intercourse obtained from the results of the study. Several patients talked about it or were worried about its occurrence because of a change in the type of sexual behavior.

“As a result of the decrease in our sexual intercourse, my husband cheat on me… after all, he is a man and his sexual needs is differs from mine… he may not bear it.” (P3).

“Six months ago, I found out that my husband was betraying me. I realize another woman in my absence when I was not there, but he wants to deny it. Still, I am sure that he is betraying me.” (p5).

### Protected sexual intercourse

Protected sex was identified as another subcategory of Adaptation to challenges of sexual intercourse in this study. The most important protective methods were the use of condoms and reducing skin contact during sex. Regarding the use of condoms, a participant stated that:

“The doctor told me it is better to use a condom so that, your husband would not get infected.” (P2).

Another method used by couples to have protected sex was reducing skin contact because; fear of transmitting the disease despite using a condom was one of the most important concerns of patients. A participant said that:

“I was just afraid that my husband would get infected. That is why I have always told him not to touch me. We have tried to have the least skin contact with the warts.” (P9).

Having sex with spouse while wearing underwear by the husband was another way to reduce the couple’s skin contact during sex. Sometimes, this method was recommended by a doctor, despite not being scientific and not having a scientific source and being solely based on experiences. In this regard, one participant who was an infectious disease specialist said that:

“This may not have been mentioned in a paper or scientific source. But, I recommend it is better to wear underwear and to tear or open underwear and also use a condom and do not cause the healthy skin of the spouse to have contact with the infected person’s warts directly.” (P19).

Sometimes, this procedure was performed by the patient himself without the doctor’s advice, as one participant said that:

“I found a solution for our problem, although it is very difficult for our skin not to come in contact with each other …. My husband wears his underwear and has sex with me.” (P13).

### Concealment of lesions

Concealment of lesions from spouse was another dimension of adaptation to challenges of sexual intercourse identified in this study. According to some patients, appearance of warts was described as hideous and this unpleasant appearance caused concern in patients. Concerns about appearance of genital warts in patients can reduce their mental image of the body. Following change in the patient’s mental image of the body, the patient’s self-confidence during sexual intercourse was found to decrease due to fear of seeing the lesions by the spouse and led to covering the lesions in different ways to hide unpleasant appearance of the wart from the spouse. A participant gave an example:

“Most of the time, during sex, I cover the wart lesions with a Band-Aid, and my husband does not tell me anything because, he knows I am sensitive … I hate seeing the lesions because of their very ugly appearance.” (P4).

Some women, fearing their husbands would see the lesions, refrained from having sex until the lesions were completely gone with one or more cryotherapy sessions.

“At first, I was anxious that my husband might see… the warts are very ugly…. but then I quickly went to the clinic and had cryotherapy done, and thank God it was removed in one session…. I didn’t have sex until the end of the week… If it happens again, I will do the same thing again… before my husband sees it.” (P13).

### Focusing on personal hygiene by couples after sexual activity

Focusing on personal hygiene by couples after sexual activity was another dimension of adaptation to challenges of sexual intercourse that almost all patients mentioned it. Many patients looked for the cause of the disease in asexual ways, such as lack of personal hygiene and identified it as the main cause of the disease. After accepting the fact that poor personal hygiene can lead to genital warts, most patients spoke about obvious changes in performing hygiene recommendations immediately after sexual intercourse.

“In the past, sometimes I wanted to sleep after having sex and I did this. But now, I force myself to go to the bathroom and wash myself immediately. I recommend the same to my husband.” (P8).

“I say to myself, surely these warts will become more and worse by not observing personal hygiene… that’s why my husband and I should take a bath and change the sheets immediately after sex… Sex has become difficult for me.” (p6).

## Discussion

This study explores women’s perceptions and experiences of sexual compatibility following genital warts. Since sexual compatibility involves emotional, cognitive, and behavioral factors during sexual intercourse that increase the couple’s satisfaction with sexual intercourse, this study showed that in most participants, genital warts had a negative effect on sexual compatibility and reduced it. In the present study, many participants stated that after being infected with genital warts, they reduced or deleted the number of sexual intercourses at their request or on the advice of a doctor. Some previous studies were consistent with our findings and reported that many participants reduced or deleted the number of sexual intercourses after HPV infection [27]. 72% of patients reduced their sexual intercourse after infection. 71% stated that the diagnosis of HPV had a negative effect on the relationship with the new sexual partner [13]. Traditional Chinese women, despite trying to maintain a marital relationship after being infected with HPV, tried to ensure that the disease did not affect their role as a wife. HPV infection reduced sexual desire in these women and reduced the number of sexual intercourses in them, and ultimately affected the quality of couples’ relationships [28]. Taberna et al. (2017) also reported that only 38% of HPV patients said that their sexual life and that of their sexual partner were not affected by HPV. Most patients reduced the number of sexual intercourse after diagnosis [29].

The present study results showed that many participants, besides changing the number of sexual intercourse, also changed the type of sexual intercourse after being infected with genital warts. These changes were especially evident in anal and oral sex. In contrast, the study of Taberna et al. (2017) showed that in patients with cervical HPV positive, oral sex at least once a week was reported in 9–10% of people, and this rate was in patients with cervical HPV negative 4–5%. The two groups were not significantly different from each other [29]. Difference between the research samples is among major reasons for the difference between our study results and this finding. We studied the change in sexual behavior in patients with genital warts, which is one of the most critical complications of HPV. As many studies have confirmed, the psychological damage of sexual genital warts is more severe.

Anxiety and worry can reduce sexual desire and thus directly reduce the couple’s sexual compatibility [12]. Most concern of participants was the fear of transmitting the disease to their spouses. This concern was especially deep in women whose husbands did not have any lesions because many participants found that the absence of lesions in their husbands was a sign of his health. Therefore, women were more anxious about transmitting the disease to their husbands during sexual intercourse. The findings of some studies are in line with this and confirm this [14, 15, 17, and 30]. Faced with this fear and concern of transmitting the disease, many participants in the present study engaged in protected sex, such as using condoms, wearing underwear during sex, and minimizing skin contact during sex. The results of the qualitative study of Lin (2011) were in line with our study, so that most patients with HPV stated they used more condoms after infection [24]. While the results of a mixed-method study by Daley (2010) et al., who assessed the psychosocial affects of HPV, disagree with our study, Daley reported that 39.2% of participants reported using condoms at all or rarely. 16.9% reported occasional use, and only 12.8% reported using condoms regularly. Although most participants considered condoms to be a suitable method of preventing disease transmission [31]. The most important reason for the difference between this finding and the present study is the difference between the type of study and the samples because the qualitative study records the experimental and perceived findings and has nothing to do with numbers. In addition, the participants in the present study were patients with genital warts. Because genital warts cause more psychological and sexual damage than HPV, these women are certainly more concerned about the spread or transmission of lesions. As a result, they place more emphasis on preventing the transmission or spread of the disease.

The present study results show that women may believe that avoiding or reducing the number of sexual intercourse after the infection also leads to increase the extramarital relationship of spouse and this vicious cycle causes women’s sexual incompatibility.

Extramarital relationship is a painful and complex phenomenon and leads to many problems such as shock, confusion, anger, depression, and impaired self-esteem in the betrayed person [32]. Despite cultural differences in different societies and the existence of different views of women on extramarital relationship, this subject is still essential for women and creates negative feelings in them.

Some previous studies confirmed that the suspicion of extramarital relationship has always been one of the main concerns of women. Also, the fear of the husband’s suspicion of the patient’s extramarital relationship was another concern expressed by patients, which is consistent with the results of the present study [24, 33]. Unfortunately, few studies assessed extramarital relationship and its effect on women’s sexual function. Perhaps the most important reason is that in most quantitative tools, this dimension of sexual, psychological effects has been ignored, and only in qualitative studies this experience of women is considered.

The present study results showed that women with genital warts have a negative image of their body because of the unpleasant appearance of the lesions, which reduces their self-confidence and indirectly has a negative effect on sexual function and sexual compatibility. So this negative self-image of the body in these women led to feelings of unattractiveness, shame, or disgust. For this reason, to solve this problem, some women tried to hide the lesions in different ways, and as a result, some of their sexual behaviors changed, willingly or unwillingly. Studies have shown that women suffer more harm than men due to negative self-image and the consequences have a more negative impact on their sexual life. In a cross-sectional study, 90% of women and 62% of men reported their self-esteem decreased. Also, 77% of women and 46% of men said the negative effects of genital warts on their sexual life [16]. The findings of a study also confirmed that women with genital wart are more vulnerable in two dimensions: body image and sexual affects [17]. Drolet et al. (2011) also reported that genital warts affect body dimensions, self-image, sexual activity, and fear of transmitting the disease to a sexual partner [14].

Many participants in the present study stated that poor personal hygiene is one cause of genital warts, so after being infected with the genital wart, they forced themselves to perform some hygiene behaviors before, during, and immediately after sex, which is definitely not to the liking of them or their spouses and thus has a negative impact on the quality of the couple’s sexual relationship.

While previous studies reported that patients with HPV expressed that Pap smear, vaccination, and condoms were appropriate prevention methods and Recommended these methods to their friends and sexual partners [24, 29]. The most important reasons for our differences with these studies are the different levels of knowledge and awareness of the participants and the cultural differences of the research environment.

Since, most of the available studies have paid attention to the psychosexual affects of HPV infection. Few studies have focused on the psychosexual affects of genital warts, most of which have been quantitative and have used standard questionnaires. And the results may not come from the feelings, emotions, and perceived experiences of the infected women, so the absence of a qualitative study in this area was quite obvious. It is hoped that the present study results will be effective as a resource for policymakers in this field, medical staff, and researchers in promoting sexual compatibility of women with genital warts.

## Limitations

The limitations of this study were the impossibility to separate women with low-risk and high-risk HPV, the impossibility to separate women who had genital wart lesions for the first time from women who had repeated recurrences, and the impossibility to study unmarried women with genital warts.

Therefore, it is suggested that in future studies, in addition to the above, a qualitative study should be considered to understand the experience of single and married men with genital warts.

## Conclusion

This study revealed the perceptions and experiences of women with genital warts about sexual compatibility. The main concepts found in this study focus on sexual intercourse-related challenges. It seems that recognizing women’s sexual compatibility challenges after getting genital warts may help take effective and practical measures to improve their sexual compatibility and health.

## Data Availability

The datasets used and/or analysed during the current study are available from the corresponding author upon reasonable request.
